# Synthesis and characterization of nanoparticle thin films of a-(PbSe)_100−*x*_Cd_*x*_ lead chalcogenides

**DOI:** 10.1186/1556-276X-8-148

**Published:** 2013-04-02

**Authors:** M A Alvi, Zishan H Khan

**Affiliations:** 1 Department of Physics, Faculty of Science, King Abdulaziz University, Jeddah 21589, Saudi Arabia; 2 Center of Nanotechnology, King Abdulaziz University, Jeddah 21589, Saudi Arabia; 3 Department of Applied Science and Humanities, Jamia Millia Islamia, New Delhi 110025, India

**Keywords:** Amorphous lead chalcogenides, Nanoparticle thin films, Raman spectra, Photoluminescence, Optical bandgap, dc conductivity

## Abstract

We report the synthesis of amorphous (PbSe)_100−*x*_Cd_*x*_ (*x* = 5, 10, 15, and 20) nanoparticle thin films using thermal evaporation method under argon gas atmosphere. Thin films with a thickness of 20 nm have been deposited on glass substrates at room temperature under a continuous flow (50 sccm) of argon. X-ray diffraction patterns suggest the amorphous nature of these thin films. From the field emission scanning electron microscopy images, it is observed that these thin films contain quite spherical nanoparticles with an average diameter of approximately 20 nm. Raman spectra of these a-(PbSe)_100−*x*_Cd_*x*_ nanoparticles show a wavelength shift in the peak position as compared with earlier reported values on PbSe. This shift in peak position may be due to the addition of Cd in PbSe. The optical properties of these nanoparticles include the studies on photoluminescence and optical constants. On the basis of optical absorption measurements, a direct optical bandgap is observed, and the value of the bandgap decreases with the increase in metal (Cd) contents in PbSe. Both extinction coefficient (*k*) and refractive index (*n*) show an increasing trend with the increase in Cd concentration. On the basis of temperature dependence of direct current conductivity, the activation energy and pre-exponential factor of these thin films have been estimated. These calculated values of activation energy and pre-exponential factor suggest that the conduction is due to thermally assisted tunneling of the carriers.

## Background

Metal chalcogenides, especially zinc, cadmium, and lead, have a lot of potential as efficient absorbers of electromagnetic radiation [1-3]. In recent years, there has been considerable interest in lead chalcogenides and their alloys due to their demanding applications as detectors of infrared radiation, photoresistors, lasers, solar cells, optoelectronic devices, thermoelectric devices, and more recently, as infrared emitters and solar control coatings [4-6]. A lot of work has also been focused on the fundamental issues of these materials possessing interesting physical properties including high refractive index [6-8].

There have been many theoretical and experimental studies on lead chalcogenides (PbS, PbSe, and PbTe) [9,10]. These chalcogenides are narrow, direct bandgap semiconductors (IV-VI groups) and crystallized at ambient condition in the cubic NaCl structure. They possess ten valence electrons instead of eight for common zinc blende and wurtzite III-V and II-VI compounds. They also exhibit some unusual physical properties, such as anomalous order of bandgaps, high carrier mobility, and high dielectric constants. All these unique properties of these semiconductors have inculcated great interest in the fundamental studies of these materials. Thin film semiconductor compounds, especially lead chalcogenide, and their alloys have drawn a lot of attention due to their technological importance and future prospects in various electronic and optoelectronic devices [11-13].

Nano-chalcogenides continue to attract the attention of researchers and engineers as a very large group of interesting solids in which unusual physical and chemical phenomena are revealed and as the materials that open new roads in science and technology. The nonlinear optical properties of these materials have attracted much attention because of their large optical nonlinearity and short response time. The size, shape, and surface characteristics have a strong influence on the physical properties of nanomaterials. Therefore, much attention has been paid in controlling these parameters to manipulate the physical properties of nanomaterials. Nanostructure formation has been explored for many kinds of materials, and this leads to an interesting topic also for lead chalcogenides. Lead chalcogenide possesses unique characteristics which are different from those in oxide and halide glasses, i.e., molecular structures and semiconductor properties. However, studies on lead chalcogenides at nanoscale are still at their early stages, and accordingly, overall features of these nanostructures have not been discovered.

Several workers reported the electrical and optical properties of PbSe in bulk form [14-17]. Many studies on PbSe films synthesized by chemical techniques are available in the literature [18-22]. There are also few reports on PbSe films and PbSe nanostructured thin films deposited by thermal evaporation technique [23-26]. Ma et al. [27] deposited polycrystalline PbSe thin films on Si substrates by thermal reduction method with carbon as the reducing agent. Kumar et al. [28] have studied the electrical, optical, and structural properties of PbSe_1−*x*_Te_*x*_ thin films prepared by vacuum evaporation technique. Lin et al. [29] reported the fabrication and characterization of IV-VI semiconductor Pb_1−*x*_Sn_*x*_Se thin films on gold substrate by electrochemical atomic layer deposition method at room temperature. Pei et al. [30] studied the electrical and thermal transport properties of lead-based chalcogenides (PbTe, PbSe, and PbS) with special emphasis on the lattice and the bipolar thermal conductivity. Gad et al. [31] have studied the optical and photoconductive properties of Pb_0.9_Sn_0.1_Se nanostructured thin films deposited by thermal vacuum evaporation and pulse laser technique.

Recently, in a joint article from one of us [32], the structural, optical, and electrical properties of polycrystalline cadmium-doped lead chalcogenide (PbSe) thin films are reported. They also studied the optical bandgap, optical constants, and temperature dependence of direct current (dc) conductivity of these thin films in polycrystalline form. In the present work, we have synthesized the materials, i.e., (PbSe)_100−*x*_Cd_*x*_ in amorphous form using melt quenching technique and the prepared thin films containing nanoparticles using thermal evaporation method. Here, all the calculated experimental parameters are reported on the amorphous thin films containing nanoparticles of (PbSe)_100−*x*_Cd_*x*_.

## Methods

The source material (PbSe)_100−*x*_Cd_*x*_ with *x* = 5, 10, 15, and 20 were synthesized by direct reaction of high purity (99.999%) elemental Pb, Se, and Cd using melt quenching technique. The desired amounts of the constituent elements were weighed according to their atomic percentage and then sealed in quartz ampoules under a vacuum of 10^−6^ Torr. The bulk samples of (PbSe)_100−*x*_Cd_*x*_ were prepared in steps. Initially, we have prepared PbSe in amorphous form, then doped with cadmium, and finally synthesized the (PbSe)_100−*x*_Cd_*x*_ in amorphous form using melt quenching. The sealed ampoules containing the samples PbSe and Cd were kept inside a programmable furnace, where the temperature was raised up to 923 K at the rate of 4 K/min and then maintained for 12 h. During the melt process, the ampoules were agitated frequently in order to intermix the constituents to ensure homogenization of the melt. The melt was then quenched rapidly in ice water.

Thin films of (PbSe)_100−*x*_Cd_*x*_ with a thickness of 20 nm were deposited on glass substrates at room temperature under argon pressure of 2 Torr using an Edward Coating Unit E-306 (Island Scientific, Ltd., Isle of Wight, England, UK). The thickness of the films was measured using a quartz crystal monitor (Edward model FTM 7). The earthed face of the crystal monitor was facing the source and was placed at the same height as the substrate. Evaporation was controlled using the same FTM 7 quartz crystal monitor.

The surface morphology of these thin films was studied by field emission scanning electron microscopy (FESEM). We have dispersed these samples in acetone solution, and a drop of solution is dispersed on carbon tape. The morphology of these dispersed particles was also studied. This suggested that the dispersed nanoparticles are aggregated with the average diameter of 20 nm. The X-ray diffraction (XRD) patterns of (PbSe)_100−*x*_Cd_*x*_ chalcogenide thin films were recorded using an X-ray diffractometer (Ultima-IV, Rigaku Corporation, Tokyo, Japan). The copper target (Cu-K*α*, *λ* = 1.5406 Å) was used as a source of X-rays. These measurements are undertaken at a scan speed of 2°/min for the scanning angle ranging from 10° to 70°. Thin films composed of nanoparticles were used for measuring optical and electrical parameters. For optical studies, we recorded the Raman spectra, photoluminescence, optical absorption, reflection, and transmission of these thin films containing nanoparticles. Optical absorption and reflection of these thin films were measured by UV–vis spectrophotometer (UV-1620PC, Shimadzu Corporation, Nakagyo-ku, Kyoto, Japan). Raman spectrum is recorded by a Raman spectrophotometer (DXR, Thermo Fisher Scientific, Waltham, MA, USA), and photoluminescence had been measured by a spectro-fluorophotometer (RF-5301PC, Shimadzu). To study the electrical transport properties, dc conductivity of these thin films was measured as a function of temperature. The resistance of these nanoparticle thin films was measured for a temperature range of 293 to 473 K. To measure the resistance, two silver thick electrodes were pasted on these thin films using silver paste. All these measurements were performed in a specially designed *I*-*V* measurement setup (4200 Keithley, Keithley Instruments Inc., Cleveland, OH, USA), which was evacuated to a vacuum of 10^−6^ Torr using a turbo molecular pump. In this setup, thin film was mounted on the sample holder with a small heater fitted below, and the temperature dependence of dc conductivity was studied.

## Results and discussion

**Figure 1:**
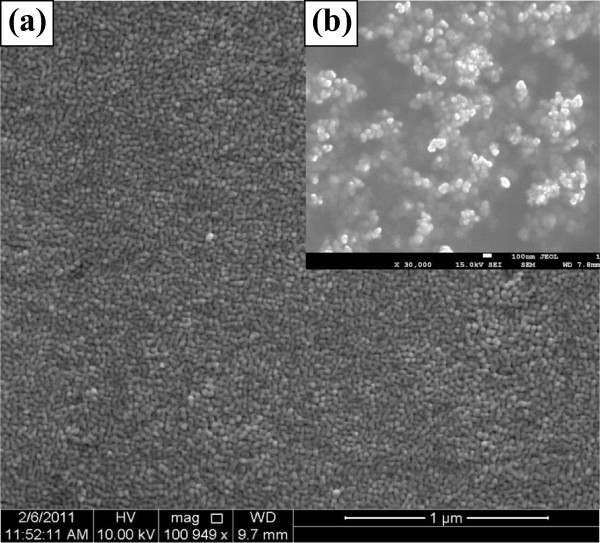
FESEM image of (a, b) thin films of a-(PbSe)_90_Cd_10_ nanoparticles.

**Figure 2:**
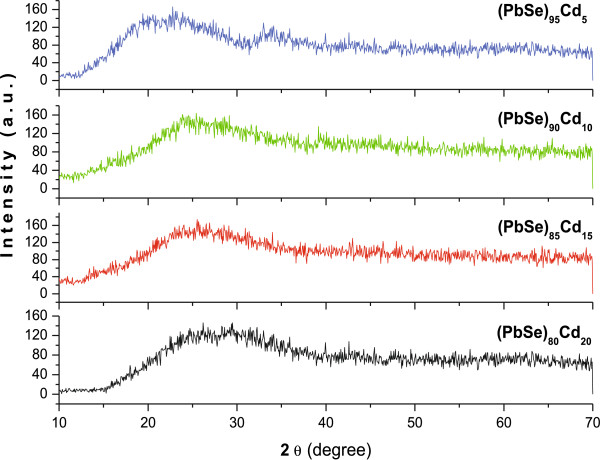
**XRD patterns at various concentrations of Cd in thin films of a-(PbSe)**_**100**−***x***_**Cd**_***x***_**nanoparticles.**

**Figure 3:**
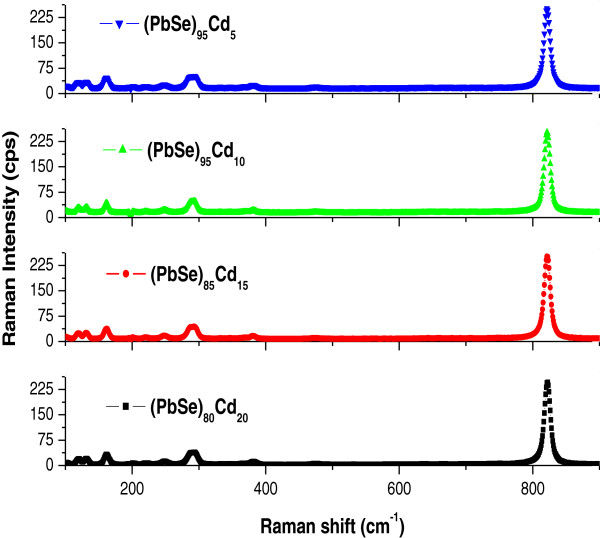
**Raman spectra at various concentrations of Cd in thin films of a-(PbSe)**_**100**−***x***_**Cd**_***x***_**nanoparticles.**

The morphological studies of these thin films show the presence of high yield of nanoparticles on the surface (Figure 1a). To understand the shape and size of these nanoparticles, we have further undertaken the morphological studies of the dispersed solution of these nanoparticles. Our studies suggest that these nanoparticles are aggregated with an average size of approximately 20 nm, and the particles are quite spherical (Figure 1b). Figure 2 presents the XRD pattern of these nanoparticle thin films. The XRD spectra do not show any significant peak for the thin films of all the studied alloy composition, thereby suggesting the amorphous nature of these nanoparticles synthesized in this study. Raman spectra of (PbSe)_100−*x*_Cd_*x*_ nanoparticles for different concentrations of cadmium are shown in Figure 3. Several Raman bands are observed at 116, 131, 162, 218, 248, 289, 383, and 822 cm^−1^. The weak peak observed at 116 cm^−1^ probably originates from the surface phonon (SP) mode, which is close to the reported value of 125 cm^−1^ for the SP mode in the case of PbSe nanoparticles [33]. The peak at around 131 cm^−1^ is assigned to the lattice mode vibration. It is an elementary transition, and the energy of this lattice phonon is 16.2 MeV. Murali et al. [33] observed a Raman peak at 135 cm^−1^ for the PbSe thin films. It is designated as lattice phonon (LO) mode. Similarly, the peaks observed at 162, 218, and 248 cm^−1^ may be attributed to 2LO(*X*), LO(*L*) + LA(*L*) and 2LO(*A*) vibration bands, respectively [34]. The peak observed at around 289 cm^−1^ is closer to the reported value of 279 cm^−1^, which is to be associated with two phonon scattering (2LO) [35]. The high-frequency peak that appeared at 822 cm^−1^ is in accordance with the polar theory, which is close to the reported value of 800 cm^−1^ for PbSe films possibly corresponding to the ground state energy of the polar on the study of Appel [36]. It is observed from the Raman studies that this alloy also contains some phases of CdSe, and a peak at 383 cm^−1^ has been observed. This peak is near the reported value of 410 cm^−1^, corresponding to the CdSe LO phonon mode [37,38]. Here, it is clear that all the observed Raman peaks show a wavelength shift on adding Cd to the PbSe system. In the case of the present system of (PbSe)_100−*x*_Cd_*x*_ nanoparticles, this shift in wavelength on low as well as on high sides may be associated with the shape of dispersion of LO phonon with a maximum wavelength at the zone center, which decreases as the phonon vector moves toward the zone edges. It is also suggested that the optical phonon line will also get broadened on reducing the size to nanoscale dimensions. This broadening may also originate from the disorder present in these nanoparticles.

**Figure 4:**
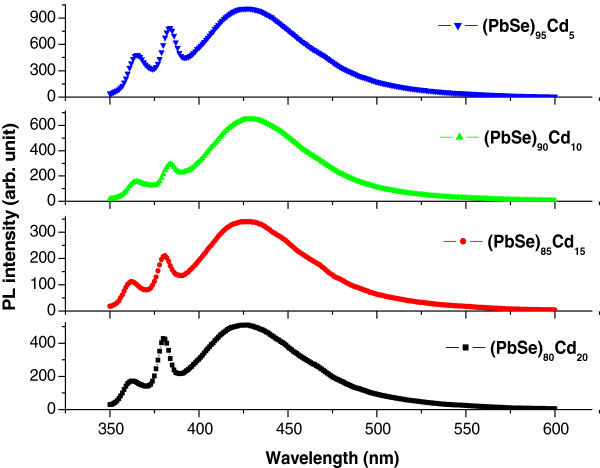
**Photoluminescence spectra at various concentrations of Cd in thin films of a-(PbSe)**_**100**−***x***_**Cd**_***x***_**nanoparticles.**

The room-temperature photoluminescence (PL) spectra of these thin films of a-(PbSe)_100−*x*_Cd_*x*_ nanoparticles as a function of incident wavelength is presented in Figure 4. The spectrum shows the emission peak under PL excitation wavelength at 300 nm within the range of 300 to 600 nm. We have observed the emission peak at 360 and 380 nm and a broad peak at 425 nm for a-(PbSe)_100−*x*_Cd_*x*_ nanoparticles. These peaks show a shift to the lower wavelength side as the metal (Cd) concentration increases. It is suggested that this shift in the emission peaks toward the lower wavelength side may be attributed to the narrowing of the bandgap of a-(PbSe)_100−*x*_Cd_*x*_ nanoparticles with the increase in cadmium concentration. This shows clearly an agreement with our results on the variation of optical bandgap with metal (Cd) content, which decreases with the increase in Cd content. It is also observed that these peaks show a broad full width at half maximum, which suggests the effect of size reduction to nanoscale in the present samples. Arivazhagan et al. [39] studied the effect of thickness on the vacuum-deposited PbSe thin film. They reported that the emission peak centered at 380, 386, 388, and 405 nm for the films of thickness 50, 100, 150, and 200 nm, respectively. This suggests that the peak shows a blueshift with the decreasing film thickness. In our case, we have deposited the films of 20-nm thickness. Therefore, the peak observed at 360 nm shows a further blueshift due to the decrease in film thickness (20 nm) as compared with that of the reported results of 50-nm-thick PbSe films. A new peak originating at 380 nm may be due to the addition of Cd to PbSe. These peaks show a blueshift with the increase in Cd content. Several workers [40] showed an emission peak at 420 nm under the PL excitation at 300 nm for nanocrystalline PbSe. In our case, we have also observed the emission peak at 425 nm for the thin films of a-(PbSe)_100−*x*_Cd_*x*_ nanoparticles, which shows a slight red shift as compared with that of the reported results. This may be due to the disorder (amorphous nature) present in the films. This peak also shows a slight blueshift with the increase in Cd content. Therefore, the peak observed at 425 nm agrees well with that of the reported results [40].

The understanding of optical and electrical processes in lead chalcogenide materials in nanoscale is of great interest for both fundamental and technological points of view. In recent years, owing to their very interesting physical properties, this particular material has raised a considerable deal of research interest followed by technological applications in the field of micro/optoelectronics. Significant research efforts have been focused to the study of the optical and electrical properties of this compound in thin film formation because the optimization of device performance requires a well-established knowledge of these properties of PbSe and metal-doped PbSe thin films. Here, we have studied the optical absorption, reflection, and transmission of amorphous thin films of (PbSe)_100−*x*_Cd_*x*_ nanoparticles as a function of the incident wavelength in the range of 400 to 1–200 nm.

The optical absorption studies of materials provide a simple approach to understand the band structure and energy gap of nonmetallic materials. Normally, the absorption coefficient is measured in the high and intermediate absorption regions to study the optical properties of materials. It is one of the most important means of determining the band structures of semiconductors. On the basis of measured optical density, we use the following relation to estimate the values of the absorption coefficient [4]:

(1) $$\mathrm{Absorption}\;\mathrm{coefficient}\;\left(\alpha \right)=\mathrm{OD}/t,$$

where OD is the optical density measured at a given layer thickness (*t*).

On the basis of the calculated values of absorption coefficient, we have observed that the value of absorption coefficient increases with the increase in photon energy for all the studied thin films of a-(PbSe)_100−*x*_Cd_*x*_ nanoparticles. During the absorption process, a photon of known energy excites an electron from a lower to a higher energy state, corresponding to an absorption edge. In the case of chalcogenides, we observe a typical absorption edge, which can be broadly attributed to one of the three processes: (1) residual below-gap absorption (2) Urbach tails, and (3) interband absorption. Highly reproducible optical edges are being observed in chalcogenide glasses. These edges in chalcogenides are relatively insensitive to the preparation conditions, and only the observable absorption [41] with a gap under equilibrium conditions accounts for the first process. A different type of optical absorption edge is observed in amorphous materials, and absorption coefficient increases exponentially with the photon energy near the energy gap. A similar behavior has also been observed in other chalcogenides [42]. This optical absorption edge is known as the Urbach edge and is given as follows:

(2) $$\mathrm{Absorption}\;\mathrm{coefficient}\;\left(\alpha \right)∼\exp\left[A\left(hv-hv_{0}\right)\right]/k_{B}T,$$

where *A* is a constant of the order of unity, *ν* is the frequency of the incident beam (*ω* = 2*πν*), *ν*_0_ is the constant corresponding to the lowest excitonic frequency, *k*_B_ is the Boltzmann constant, and *T* is the absolute temperature.

The calculated values of the absorption coefficient for thin films of a-(PbSe)_100−*x*_Cd_*x*_ nanoparticles are of the order of approximately 10^5^ cm^−1^, which is consistent with the reported results [43,44]. The calculated values of absorption coefficient (*α*) are given in Table 1. It is observed that *α* shows an overall increasing trend with the increase in the metal (Cd) concentration. It is suggested that bond breaking and bond rearrangement may take place when there is increasing cadmium concentration, which results in the change in local structure of these lead chalcogenide nanoparticles. This includes subtle effects such as shifts in the absorption edge, and more substantial atomic and molecular reconfiguration which is associated with changes in the absorption coefficient and absorption edge shift.

**Table 1:** **Electrical and optical parameters in (PbSe)**_**100**−***x***_**Cd**_***x ***_**nanoparticle thin films**

**Sample**	***σ***_**dc **_**(Ω**^**−1 **^**cm**^**−1**^**) at 380 K**	***σ***_**0 **_**(Ω**^**−1 **^**cm**^**−1**^**)**	**Δ*E***_**c **_**(eV)**	**Δ*E***_**g **_**(eV)**	***α *(cm**^**−1**^**) (10**^**5**^**)**	***n *at 590 nm**	***k *at 590 nm**
(PbSe)_95_Cd_5_	3.21 × 10^-6^	2.69 × 10^8^	0.99	2.41	1.02	1.65	0.117
(PbSe)_90_Cd_10_	1.85 × 10^-6^	3.61 × 10^6^	0.91	2.19	2.36	1.83	0.632
(PbSe)_85_Cd_15_	2.64 × 10^-5^	8.62 × 10^6^	0.87	2.12	1.94	2.44	0.524
(PbSe)_80_Cd_20_	6.69 × 10^-5^	2.21 × 10^7^	0.85	2.03	3.11	2.73	0.923

In the case of amorphous semiconductors, the fundamental absorption edge follows an exponential law. Above the exponential tail, the absorption coefficient obeys the following equation [4]:

(3) $${\left(\alpha hv\right)}^{1/m}=B\left(hv-E_{g}\right),$$

where *B* is a constant, *E*_g_ is the optical bandgap, and *m* is a parameter that depends on both the type of transition (direct or indirect) and the profile of the electron density in the valence and conduction bands. The values of *m* can be assumed to be 1/2, 3/2, 2, and 3, depending on the nature of electronic transition responsible for the absorption: *m* = 1/2 for allowed direct transition, *m* = 3/2 for forbidden direct transition, *m* = 2 for allowed indirect transition, and *m* = 3 for forbidden indirect transition.

The present systems of a-(PbSe)_100−*x*_Cd_*x*_ obey the role of direct transition, and the relation between the optical gap, absorption coefficient *α*, and the energy (h*ν*) of the incident photon is given as follows:

(4) $${\left(ahv\right)}^{2}=B\left(hv-Eg\right).$$

**Figure 5:**
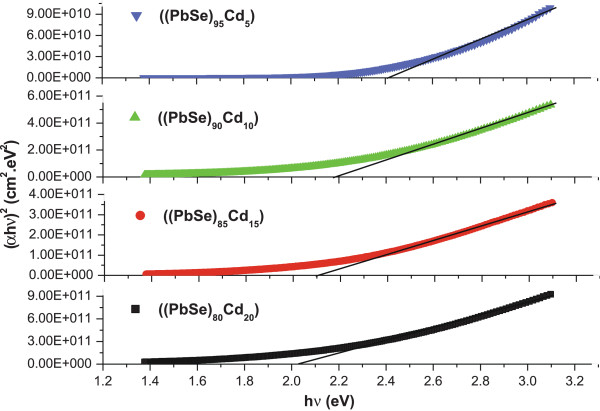
**Temperature dependence of dc conductivity.** It is in the range of 297 to 400 K at various concentrations of Cd in thin films of a-(PbSe)_100−*x*_Cd_*x*_ nanoparticles.

The variations of (*α*h*ν*)^2^ with photon energy (h*ν*) for a-(PbSe)_100−*x*_Cd_*x*_ nanoparticle films are shown in Figure 5. Using this figure, the intercept on the *x*-axis gives the value of direct optical bandgap *E*_g_, and the calculated values of *E*_g_ for a-(PbSe)_100−*x*_Cd_*x*_ nanoparticles are given in Table 1. It is clear from the table that *E*_g_ decreases with the increase in Cd concentration in this system of nanoparticles. This decrease in optical bandgap may be explained on the basis of ‘density of state model’ proposed by Mott and Davis [45]. According to this model, the width of the localized states near the mobility edges depends on the degree of disorder and defects present in the amorphous structure. In particular, it is known that unsaturated bonds together with some saturated bonds are produced as the result of an insufficient number of atoms deposited in the amorphous film [46]. The unsaturated bonds are responsible for the formation of some defects in the films, producing localized states in the amorphous solids. The presence of high concentration of localized states in the band structure is responsible for the decrease in optical bandgap on increasing dopant (Cd) concentration in these amorphous films of (PbSe)_100−*x*_Cd_*x*_ nanoparticles. This decrease in optical bandgap may also be due to the shift in the Fermi level whose position is determined by the distribution of electrons over the localized states [47].

The values of refractive index (*n*) and extinction coefficient (*k*) have been calculated using the theory of reflectivity of light. According to this theory, the reflectance of light from a thin film can be expressed in term of the Fresnel's coefficient. The reflectivity [48-50] on an interface is given as follows:

(5) $$n=\left[\left(1+R\right)+{\left\{{\left(1+R\right)}^{2}-{\left(1-R\right)}^{2}\left(1+k^{2}\right)\right\}}^{1/2}\right]/\left(1-R\right)$$

where the value of *k* has been calculated by using the following formula:

(6) $$k=\left(\alpha \lambda \right)/\left(4\pi \right),$$

with λ is the wavelength.

**Figure 6:**
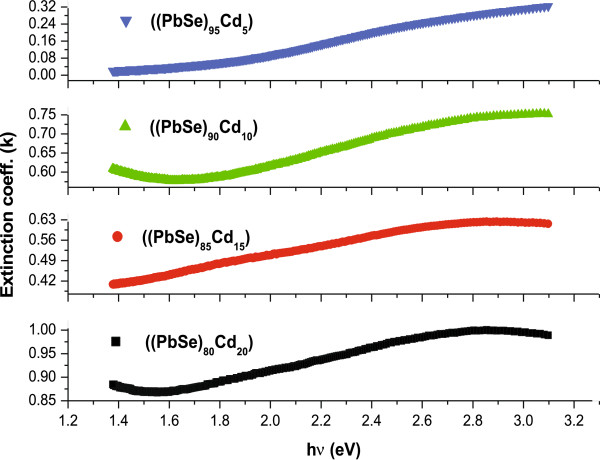
**(*α*h*ν*)**^**2 **^**against photon energy (h*ν*) for thin films of a-(PbSe)**_**100**−***x***_**Cd**_***x***_**nanoparticles.**

**Figure 7:**
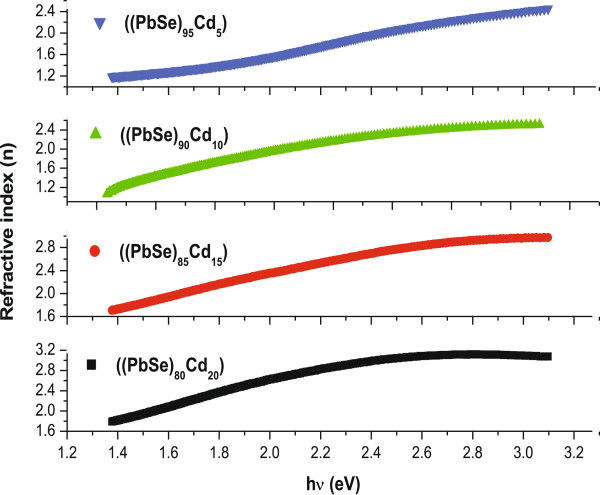
**Variation of extinction coefficient (*k*) with incident photon energy (h*ν*) in thin films of a-(PbSe)**_**100**−***x***_**Cd**_***x***_**nanoparticles.**

Figures 6 and 7 show the spectral dependence of the extinction coefficient and refractive index for a-(PbSe)_100−*x*_Cd_*x*_ thin films. It is observed that the values of these optical constants (*n* and *k*) increases with the increase in photon energy. A similar trend has also been observed for thin films of other various amorphous semiconductors [51,52]. The values of *n* and *k* for different concentrations of Cd are given in Table 1. It is evident from the table that, overall, the value of these optical constants increases with the increase in dopant concentration. This can be understood on the basis of density of defect states. It is well known that chalcogenide thin films contain a high concentration of unsaturated bonds or defects. These defects are responsible for the presence of localized states in the amorphous bandgap [53]. In our case, the addition of Cd in the PbSe alloy results in the increased number of unsaturated defects. Due to this increase in the number of unsaturated defects, the density of localized states in the band structure increases, which consequently leads to the increase in values of refractive index and extinction coefficient with the addition of metal (Cd) content.

For the study of electrical transport in amorphous semiconductors, especially chalcogenide glasses, dc conductivity is one of the important parameters. The dc conductivity of chalcogenide glasses depends on the combination of starting components, synthesis conditions, rate of melt annealing, purity of starting components, thermal treatment, and on some other important factors. The electrical conduction process in amorphous semiconductors is generally governed by the three mechanisms namely (1) the transfer of charge carriers between delocalized states in the conduction band (*E* >*E*_c_) and valence band (*E* <*E*_v_), (2) transitions of charge carriers in the band tails, and (3) the hopping of charge carriers between delocalized states in bands near the Fermi level (*E*_F_). To explain the conduction mechanism in amorphous semiconductors, studies on temperature dependence of conductivity is reported by various workers [54-57]. It is understood that conduction in chalcogenide glasses is intrinsic [58,59] and that the Fermi level is close to the midway of the energy gap. Intrinsic conduction of amorphous semiconductors is determined by carrier hopping from the states close to the edge of the valence band to localized states near the Fermi level or from the state near the Fermi level to the conduction band. The suitable conduction mechanism is decided depending on the predominant process. In the case of chalcogenide glasses, the Fermi level is somewhat shifted from the middle of the energy gap toward the valence band [60].

In the present work, we have also studied the temperature dependence of dc conductivity of thin films of a-(PbSe)_100−*x*_Cd_*x*_ nanoparticles over the temperature range of 297 to 400 K. From the variations of dc conductivity with temperature, it is found that the experimental data for the entire temperature range is fitted well with the thermally activated process model. To elucidate the conduction mechanism in the present sample of a-(PbSe)_100−*x*_Cd_*x*_ nanoparticles, we have applied the thermally activated process for the temperature region of 297 to 400 K.

**Figure 8:**
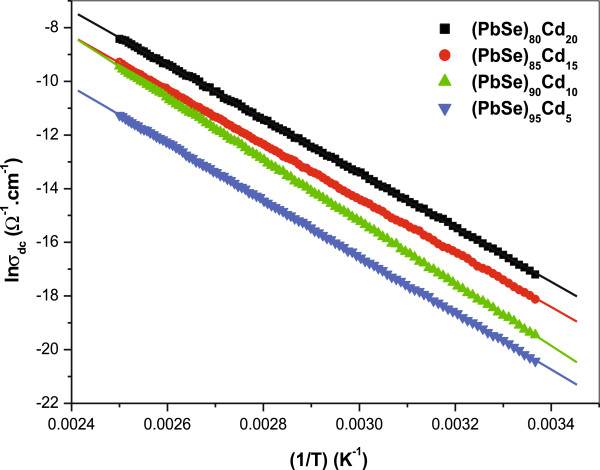
**Variation of refractive index (*n*) with incident photon energy (h*ν*) in thin films of a-(PbSe)**_**100**−***x***_**Cd**_***x***_**nanoparticles.**

The plot of ln σ_dc_ versus 1000/*T* for the temperature range of 297 to 400 K is presented in Figure 8. The graph is a straight line, indicating that the conduction in this system is through a thermally activated process. The conductivity is, therefore, expressed by the usual relation given as follows [4]:

(7) $$\sigma_{\mathrm{dc}}=\sigma_{0}\exp\left(-\Delta E_{c}/k_{B}T\right),$$

where σ_0_ represents the pre-exponential factor, and Δ*E*_c_ is the dc activation energy which is calculated from the slope of ln σ_dc_ versus 1000/*T* plot.

Using the slope and intercept of Figure 8, we have calculated the value of Δ*E*_c_ and σ_0_, respectively. The calculated values of Δ*E*_c_ and σ_0_ for different compositions of cadmium in a-(PbSe)_100−*x*_Cd_*x*_ nanoparticle thin films are shown in Table 1. On the basis of these calculated values, it may be suggested that the conduction is due to the thermally assisted tunneling of charge carriers in the extended states for the temperature range of 297 to 400 K of our sample a-(PbSe)_100−*x*_Cd_*x*_ nanoparticles. However, it is important to mention that activation energy alone does not provide any information as to whether conduction takes place in the extended states above the mobility edge or by hopping in the localized states. This is due to the fact that both of these conduction mechanisms may take place simultaneously. The activation energy in the former case represents the energy difference between mobility edge and the Fermi level, *E*_c_ − *E*_F_ or *E*_F_ − *E*_V_, and in the latter case, it represents the sum of the energy separation between the occupied localized states and the separation between the Fermi level and the mobility edge. It is evident from Table 1 that dc conductivity increases as the concentration of Cd increases, whereas the value of activation energy decreases with the increase in Cd contents in our lead chalcogenide nanoparticles. An increase in dc conductivity with a corresponding decrease in activation energy is found to be associated with a shift of the Fermi level for the impurity-doped chalcogenide [46,61]. It also shows that the Fermi level changes after the incorporation of Cd. However, it has also been pointed out that the increase in conductivity could be caused by the increase in the portion of hopping conduction through defect states associated with the impurity atoms [62].

A clear distinction between these two conduction mechanisms can be made on the basis of the pre-exponential factor value. For conduction in extended states, the value of σ_0_ reported for a-Se and other Se alloys in thin films is of the order 10^4^ Ω^−1^ cm^−1^[62]. In the present sample of a-(PbSe)_100−*x*_Cd_*x*_ nanoparticles, the value of σ_0_ is of the order 10^7^ Ω^−1^ cm^−1^. Therefore, extended state conduction is most likely to take place. An overall decrease in the value of σ_0_ is observed with the increase in Cd contents in the PbSe system, which may be explained using the shift of Fermi level on adding Cd impurity. Therefore, the decrease in the value of σ_0_ may be due to the change in Fermi level on adding Cd in the PbSe System.

## Conclusions

Thin films of amorphous (PbSe)_100−*x*_Cd_*x*_ nanoparticles have been synthesized using thermal evaporation technique. The average diameter of these nanoparticles is approximately 20 nm. Raman spectra of these a-(PbSe)_100−*x*_Cd_*x*_ nanoparticles revealed the presence of PbSe phases in as-synthesized thin films, and the observed wavelength shift in the peak position as compared with that of reported values on PbSe may be due to the addition of Cd impurity. PL spectra suggest that the peaks show a shift to the lower wavelength side as the metal (Cd) concentration increases, which may be attributed to the narrowing of the bandgap of a-(PbSe)_100−*x*_Cd_*x*_ nanoparticles with the increase in cadmium concentration. A direct optical bandgap is observed, which decreases on increasing cadmium concentration. This may also be due to the increase in the density of defect states, which results in the extension of tailing of bands. The value of refraction index and extinction coefficient increases with increasing photon energy for all samples of a-(PbSe)_100−*x*_Cd_*x*_. From temperature dependence of dc conductivity measurements, it may be concluded that conduction is taking place through the thermally activated process over the entire range of investigation. The pre-exponential factor shows an overall decreasing trend with increasing Cd content. The decrease in σ_0_ may be due to the change in the Fermi level on the addition of Cd in the lead chalcogenide system. Finally, the suitability of these nanoparticles of lead chalcogenides for various applications especially in solar cells can be understood on the basis of these properties.

## Competing interests

The authors declare that they have no competing interests.

## Authors’ contributions

Both authors - MAA and ZHK - participated equally in the experiments performed to accomplish this work and in the preparation of this manuscript. Both authors read and approved the final manuscript.

## References

[B1] Mahapatra PK Roy CB Photoelectrochemical cells with mixed polycrystalline n-type CdS-PbS and CdS-CdSe electrodes Electrochem Acta 1984 8 1435 10.1016/0013-4686(84)87023-1

[B2] Kenawy MA Zayed HA Ibrahim AM Structural, electrical and optical properties of ternary CdS_*x*_Se_1−*x*_ thin films Indian J Pure & Appl Phys 1991 8 624

[B3] Deshmukh LP More BM Holikatti SG Preparation and properties of (CdS)_*x*_-(PbS)_1−*x*_ thin-film composites Bull Mater Sci 1994 8 455 10.1007/BF02757889

[B4] Al-Ghamdi AA Al-Heniti S Khan SA Structural, optical and electrical characterization of Ag doped lead chalcogenide (PbSe) thin films J Luminescence 2013 8 295

[B5] Nair PK Garcia VM Hernandez AB Nair MTS Photoaccelerated chemical deposition of PbS thin films: novel applications in decorative coatings and imaging techniques J Phys D: Appl Phys 1991 8 1466 10.1088/0022-3727/24/8/036

[B6] Schluter M Martinez G Cohen ML Pressure and temperature dependence of electronic energy levels in PbSe and PbTe Phys Rev B 1975 8 650 10.1103/PhysRevB.12.650

[B7] Yuan S Krenn H Springholz G Bauer G Dispersion of absorption and refractive index of PbTe and Pb_1−*x*_Eu_*x*_Te (*x* < 0.05) below and above the fundamental gap Phys Rev B 1993 8 7213 10.1103/PhysRevB.47.7213 10004719

[B8] Nimtz G Schlicht B Narrow-gap lead salts Narrow-Gap Semiconductors 1983 New York: Springer-Verlag 98

[B9] Chesnokova DB Moshnikov VA Gamarts AE Maraeva EV Aleksandrova OA Kuznetsov VV Structural characteristics and photoluminescence of Pb_1−*x*_Cd_*x*_Se (*х* = 0–0.20) layers J Non-Crystt Solids 2010 8 2010 10.1016/j.jnoncrysol.2010.05.025

[B10] Bencherif Y Boukra A Zaoui A Ferhat M Lattice dynamics study of lead chalcogenides Infrared Phys Tech 2011 8 39 10.1016/j.infrared.2010.11.001

[B11] Henini M Rodgers PJ Crump PA Gallagher BL Hill G Growth and electrical transport properties of very high mobility two‐dimensional hole gases displaying persistent photoconductivity Appl Phys Lett 2054 8 65

[B12] Zogg H Alchalabi K Zimin D Kellermann K Electrical and optical properties of PbTe p-n junction infrared sensors Infrared Phys Technol 2002 8 251 10.1016/S1350-4495(02)00148-2

[B13] Kumar S Lal B Aghamkar P Husain M Influence of sulfur, selenium and tellurium doping on optical, electrical and structural properties of thin films of lead salts J Alloys Compd 2009 8 334 10.1016/j.jallcom.2009.08.126

[B14] Volkov BA Ryabova LI Khokhlov DR Mixed-valence impurities in lead telluride-based solid solutions Physics-Uspekhi 2002 8 8 819 10.1070/PU2002v045n08ABEH001146

[B15] Rogacheva EI Krivulkin IM Nashchekina ON Sipatov AY Volobuev VV Dresselhaus MS Effect of oxidation on the thermoelectric properties of PbTe and PbS epitaxial films Appl Phys Lett 2001 8 1661 10.1063/1.1355995

[B16] Humprey JN Prtriz RL Photoconductivity of lead selenide: theory of the mechanism of sensitization Phys Rev 1957 8 1736 10.1103/PhysRev.105.1736

[B17] Vurgaftman I Meyer JR Ram-Mohan LR Band parameters for III–V compound semiconductors and their alloys J Appl Phys 2001 8 5815 10.1063/1.1368156

[B18] Streltsov EA Osipovich NP Ivashkevich LS Layakhov AS Sviridov VV Electrochemical deposition of PbSe films Electrochim Acta 1998 8 869

[B19] Biro LP Candea RM Borodi G Darabont A Fitori P Bratu I Amorphous PbSe films: growth and properties Thin Solid Films 1988 8 303 10.1016/0040-6090(88)90701-8

[B20] Hankare PP Delekar SD Bhuse VM Garadkar KM Sabane SD Gavali LV Synthesis and characterization of chemically deposited PbSe thin films Mater Chem Phys 2003 8 505 10.1016/S0254-0584(03)00375-4

[B21] Grozdanov I Najdoski M Dey SK A simple solution growth technique for PbSe thin films Mater Letts 1999 8 28 10.1016/S0167-577X(98)00127-X

[B22] Molin AN Dikusar AI Electrochemical deposition of PbSe thin films from aqueous solutions Thin Solid Films 1995 8 3 10.1016/0040-6090(95)06548-2

[B23] Munoz A Melendez J Torquemada MC Rodrigo MT Cebrian J De Castro AJ PbSe photodetector arrays for IR sensors Thin Solid Films 1998 8 425 10.1016/S0040-6090(97)00576-2

[B24] Shandalova M Dashevsky Z Golana Y Microstructure related transport phenomena in chemically deposited PbSe films Mater Chem Phys 2008 8 132 10.1016/j.matchemphys.2008.05.040

[B25] Kumar S Khan ZH Khan MAM Husain M Studies on thin films of lead chalcogenides Curr Appl Phys 2005 8 561 10.1016/j.cap.2004.07.001

[B26] Li JQ Li SP Wang QB Wang L Liu FS Ao WQ Synthesis and thermoelectric properties of the PbSe_1−*x*_Te_*x*_ alloys J Alloys and Compds 2011 8 4516 10.1016/j.jallcom.2011.01.033

[B27] Ma DW Cheng C Preparations and characterizations of polycrystalline PbSe thin films by a thermal reduction method J Alloys Compds 2011 8 6595 10.1016/j.jallcom.2011.03.100

[B28] Kumar S Husain M Sherma TP Husain M Characterization of PbSe_1−*x*_Te_*x*_ thin films J Phys Chem Solids 2003 8 367 10.1016/S0022-3697(01)00252-9

[B29] Lin S Zhang X Shi X Wei J Lu D Zhang Y Kou H Wang C Nanoscale semiconductor Pb_1−*x*_Sn_*x*_Se (*x* = 0.2) thin films synthesized by electrochemical atomic layer deposition Appl Surf Sci 2011 8 5803 10.1016/j.apsusc.2011.01.108

[B30] Pei YL Liu Y Electrical and thermal transport properties of Pb-based chalcogenides: PbTe, PbSe, and PbS J Alloys and Compds 2012 8 40

[B31] Gad S Rafea MA Badr Y Optical and photoconductive properties of Pb_0.9_Sn_0.1_Se nano-structured thin films deposited by thermal vacuum evaporation and pulsed laser deposition J Alloys and Compds 2012 8 101

[B32] Khan SA Khan ZH El-Sebaii AA Al-Marzouki FM Al-Ghamdi AA Structural, optical and electrical properties of cadmium-doped lead chalcogenide (PbSe) thin films Physica B 2010 8 3384 10.1016/j.physb.2010.05.009

[B33] Murali KR Ramanathan P Characteristics of slurry coated lead selenide films Chalcogenide Letts 2009 8 3 91

[B34] Manciu FS Sahoo Y Carreto F Prasad PN Size-dependent Raman and infrared studies of PbSe nanoparticles J Raman Spectrosc 2008 8 1135 10.1002/jrs.1946

[B35] Li KW Meng XT Liang X Wang Yan H Electrodeposition and characterization of PbSe films on indium tin oxide glass substrates J Solid State Electrochem 2006 8 48 10.1007/s10008-005-0660-z

[B36] Appel J Polarons Solid State Physics, Advances in Research and Applications 1968 8 193

[B37] Ichimura M Takeuchi K Nakamura A Arai E Photochemical deposition of Se and CdSe films from aqueous solutions Thin Solid Films 2001 8 157 10.1016/S0040-6090(00)01826-5

[B38] Fomin VM Pokatilov EP Devreese JT Klimin SN Gladilin VN Balaban SN Multiphonon photoluminescence and Raman scattering in semiconductor quantum dots Solid State Electron 1998 8 1309 10.1016/S0038-1101(98)00022-7

[B39] Arivazhagan V Parvathi MM Rajesh S Impact of thickness on vacuum deposited PbSe thin films Vacuum 2012 8 8 1092 10.1016/j.vacuum.2011.10.008

[B40] Li Z Wu C Liu Y Liu T Zheng J Wu M Preparation of PbSe nanoparticles by electron beam irradiation method Bulletin of Materials Sciences 2008 8 825 10.1007/s12034-008-0131-0

[B41] Tauc J Tauc J Optical properties of amorphous semiconductors Amorphous and Liquid Semiconductors 1974 London: Plenum Press 159

[B42] Urbach F The long-wavelength edge of photographic sensitivity and of the electronic absorption of solids Phys Rev 1953 8 1324

[B43] Ilyas M Zulfequar M Husain M Optical investigation of a-Ga_*x*_Se_100−*x*_ thin films J Modern Optics 2000 8 663

[B44] Maan AS Goyal DR Sharma SK Sharma TP Investigation of electrical conductivity and optical absorption in amorphous In_*X*_Se_100−*X*_ alloys J Physique III 1994 8 493 10.1051/jp3:1994141

[B45] Mott NF Davis EA Optical properties of amorphous arsenic and the density of states in the bands Electronics Processes in Non-Crystalline Materials 1979 Oxford: Clarendon 426

[B46] Theye ML Proceedings of the 5th International Conference on Amorphous and Liquid Semiconductors 1973 1 Germany: Garmisch-Partenkirchen

[B47] Al-Agel FA Khan SA Khan ZH Zulfequar M Influence of laser-irradiation on structural and optical properties of phase change Ga_25_Se_75−*x*_Te_*x*_ thin films Mat Lett 2012 8 424 426

[B48] Khan ZH Zulfequar M Sharma TP Husain M Optical properties of a-Ga_20_Se_80−*x*_Sb_*x*_ thin films J Opt Mater 1996 8 139 10.1016/0925-3467(96)00044-4

[B49] Khan ZH Khan SA Salah N Habib S Effect of composition on electrical and optical properties of thin films of amorphous Ga_*x*_Se_100−*x*_ nanorods Nanoscale Res Letters 2010 8 1512 10.1007/s11671-010-9671-5 PMC2920423 20730131

[B50] Khan ZH Husain M Electrical and optical properties of thin film of a-Se_70_Te_30_ nanorods J Alloy and Compd 2009 8 774 10.1016/j.jallcom.2009.07.049

[B51] Khan ZH Khan SA Salah N Habib S Al-Ghamdi AA Electrical and optical properties of a-Se_*x*_Te_100–*x*_ thin films Optics & Laser Tech 2012 8 6 10.1016/j.optlastec.2011.05.001

[B52] Khan ZH Al-Ghamdi AA Khan SA Habib S Salah N Morphology and optical properties of thin films of Ga_*x*_Se_100−*x*_ nanoparticles Nanoscience and Nanotechnology Letts 2011 8 319 323 10.1166/nnl.2011.1188

[B53] Al-Hazmi FS Optical changes induced by laser–irradiation on thin films of Se_75_S_15_Ag_10_ chalcogenide Chalcogenide Letters 2009 8 63

[B54] Khan ZH Zulfeqaur M Ilyas M Husain M Non-isothermal electrical conductivity and thermo-electric power of a-Se_80−*x*_Ga_20_Te_*x*_ thin films Acta Physica Polonica (A) 2000 8 93

[B55] Khan ZH Khan SA Salah N Al-Ghamdi AA Habib S Electrical properties of thin films of a-Ga_*x*_Te_100−*x*_ composed of nanoparticles Phil Mag Letts 2011 8 207

[B56] Khan ZH Zulfequar M Malik MM Husain M Effect on Sb on transport properties of a-Se_80−*x*_Ga_20_Sb_*x*_ thin films Jap J Applied Physics 1998 8 23 10.1143/JJAP.37.23

[B57] Khan ZH Salah N Habib S Electrical transport of a-Se_87_Te_13_ nanorods J Experimental Nanoscience 2011 8 337 10.1080/17458080.2010.497946

[B58] Minaev VS Vitreous Semiconducting Alloys 1991 Moscow: Metallurgiya (in Russian)

[B59] Kostylev SA Shkut VA Himinets VV Structure, physico-chemical properties and applications of non-crystalline semiconductors Proc Int Conf Amorph Semic 1980 8 277

[B60] Feltz A Amorphous and Glassy Inorganic Solids (in Russian) 1986 Moscow: Mir Publishers, [original German edition: Amorphe und glasartige anorganische Festko¨rper. Berlin: Akademie-Verlag; 1983]

[B61] Kolomiets BT Lebedev EA Taksami IA Mechanism of the breakdown in films of glassy chalcogenide semiconductors Sov Phys Semicond 1969 8 267

[B62] Okano S Suzuki M Imura K Fukada N Hiraki A Impurity effects of some metals on electrical properties of amorphous As_2_Se_1_Te_2_ films J Non-Crys Solids 1983 8 969

